# A Case of Anti-Leucine-Rich Glioma-Inactivated Protein 1 (Anti-LGI1) Limbic Encephalitis With Normal Imaging

**DOI:** 10.7759/cureus.62387

**Published:** 2024-06-14

**Authors:** Kyle Zatyko, Yohan Kim, Muhammad S Abdullah, Andres Saenz

**Affiliations:** 1 Neurology, University of Texas (UT) Health San Antonio, San Antonio, USA

**Keywords:** autoimmune neurologic disorder, autoimmune encephalitis, limbic encephalitis, lgi-1, anti-lgi1 limbic encephalitis, anti-lgi1 le, lgi1 antibody autoimmune encephalitis

## Abstract

Anti-leucine-rich glioma-inactivated 1 limbic encephalitis (anti-LGI1 LE) is a rare autoimmune limbic encephalitis with a potentially misleading presentation that can delay diagnosis and treatment. The incremental progression of widely variable symptoms with a prominent behavioral disturbance can conceal the disease and prompt an initial psychiatric diagnosis. Although specific MRI findings ought to be evident by the time the disease progresses to frank limbic encephalitis, it appears inconsistent and ill-defined and is thus unreliable. Nevertheless, brain imaging remains prominent in the discussion, even included in some guidelines for diagnosing anti-LGI1 LE. Here, we present a case of a patient who presented after a suicide attempt with a long history of psychiatric issues, aberrant “spasms,” and subsequently encephalopathy, who was eventually diagnosed with anti-LGI1 LE only after delayed CSF antibodies studies. In this patient, symptoms emerged over two years, with multiple brain MRIs being negative, including the one completed during the hospital admission in focus.

The purpose of this case report is to encourage maintaining a broad differential when patients present with bizarre symptoms. This report underlies the importance of thorough clinical evaluation, utilization of multiple diagnostic resources, and the need for heightened awareness among healthcare providers about the subtleties of autoimmune encephalitis presentations. With anti-LGI1 LE already being severely underdiagnosed, it is important to continue reviewing various cases of patients who are diagnosed with anti-LGI1 LE and further review to understand its pathophysiology and common clinical presentation. This case also underscores the ongoing evolution in understanding anti-LGI1 LE and highlights that patients may present with unfamiliar symptoms or diagnostic challenges. The overall objective is to help providers recognize anti-LGI1 LE earlier, so treatment can be initiated sooner, leading to a better prognosis for patients.

## Introduction

Anti-leucine-rich glioma-inactivated 1 limbic encephalitis (anti-LGI1 LE) is a subtype of autoimmune limbic encephalitis. It is associated with antibodies targeting neuronal cell surface and synaptic proteins. The LGI1 antibody is believed to prevent the binding of LGI1, a glycoprotein within the synaptic space that is important to synaptic transmission, to its many targets causing disruptions of cellular currents and communication [1]. These disruptions increase the firing tonicity, thus potentially inducing epileptiform activity [2]. The presentation of LGI1 encephalitis varies across patients, but the most common symptoms are epilepsy, faciobrachial dystonic seizures (FBDS), cognitive dysfunction, neuropsychiatric disturbances, sleep disorders, hyponatremia, and disturbance of consciousness [3]. Neuropsychiatric disturbances in the initial phase, followed by seizures or FBDS, have been shown to precede the development of frank limbic encephalitis [4]. Cases of anti-LGI1 LE typically display hyperintensities on brain MRI on the T2, FLAIR, and DWI sequences typically within the mesial temporal lobe and, less commonly, the basal ganglia, especially when symptoms have been a chronic process [5]. The diagnosis of anti-LGI1 LE is confirmed with positive serum or CSF titers for the anti-LGI1 antibody. Treatment in the acute setting entails high-dose steroids and possible plasma exchange or intravenous immunoglobulin for more severe cases, with patients usually going on long-term disease-modifying antirheumatic drugs for chronic management.

This case presents a challenging and unique patient who was misdiagnosed for years due to multiple normal MRI brain images despite having such chronic and profound yet vague symptoms of anti-LGI1 LE. It is rare for patients with such a long-term course of anti-LGI1 LE symptoms, such as FBDS and neurocognitive dysfunction, to have benign imaging. Overall, this is significant because by broadening the diagnostic approach and considering rare entities like anti-LGI1 LE, physicians can minimize the patient’s risk of long-term irreversible cognitive sequelae and promote more favorable outcomes through earlier detection and targeted treatment.

## Case presentation

Our case presents a 62-year-old male patient with a history of hypertension, type 2 diabetes, depression, and presumed schizophrenia (visual and auditory hallucinations) with prior hospitalizations over the years for several progressive neuropsychiatric issues. His baseline psychiatric disorders were reportedly stable on aripiprazole and bupropion, and the patient was on Keppra and Vimpat upon the presenting hospital encounter for a prior history of presumed seizures.

In the past, the patient had multiple hospital admissions for unexplained neurological deficits and psychiatric complaints. For instance, the patient was admitted to an outside hospital for acute onset stroke-like symptoms, including left facial weakness, slurred speech, left-sided paresthesia, and generalized weakness, and was found to be concurrently experiencing hallucinations, abnormal behaviors, and insomnia. Workup, including EEG and MRI imaging at that time, was inconclusive.

On another prior hospitalization, the patient came in with “spasms” of the face and left upper extremity. During that presentation, there was no inciting factor, and it was documented that the patient did not have any recent illnesses, lack of sleep, drug or alcohol usage, or delineation of his usual lifestyle. He was also found to have increased difficulty concentrating, erratic behaviors, and increased insomnia leading up to the specific hospital admission. His vital signs were all within normal range, but the initial labs showed a mild hyponatremia of 130; otherwise, metabolic and infectious panels were unremarkable, and alcohol and drug screening were negative. The patient then had an EEG that was only prominent for right temporal slowing. The concurrent neuroimaging, including CT head and MRI brain, were unrevealing for acute processes. The patient’s symptoms subsided without management, so a lumbar puncture was deferred. After hospital discharge, the patient continued to have jerking-like episodes leading to multiple visits to the emergency room and other hospital admissions, in which he was started on Keppra and Vimpat prior to this presenting case.

During this presenting hospitalization, the patient was initially admitted to the psychiatric wards for new-onset suicide ideation. His vital signs were again within normal range, and the patient's admission lab workup was relevant for mild hyponatremia, elevated glucose, and a urinary tract infection, for which he was started on antibiotics. While admitted to the hospital, he developed two breakthrough generalized tonic-clonic seizures and was transferred to the neurology service for further management. On evaluation, the patient had marked worsening of his neuropsychiatric issues with severe disorientation and inability to speak sentences. The family reported a change from his baseline mental status and complaints of inability to sleep throughout the night. Meanwhile, he had intermittent myoclonic jerks involving his left face and left arm. Along with the myoclonic jerks and increased psychiatric symptoms, throughout the hospital course, the patient developed autonomic imbalances, including both hypo and hyperthermia, tachycardia, and hypotension.

**Figure 1:**
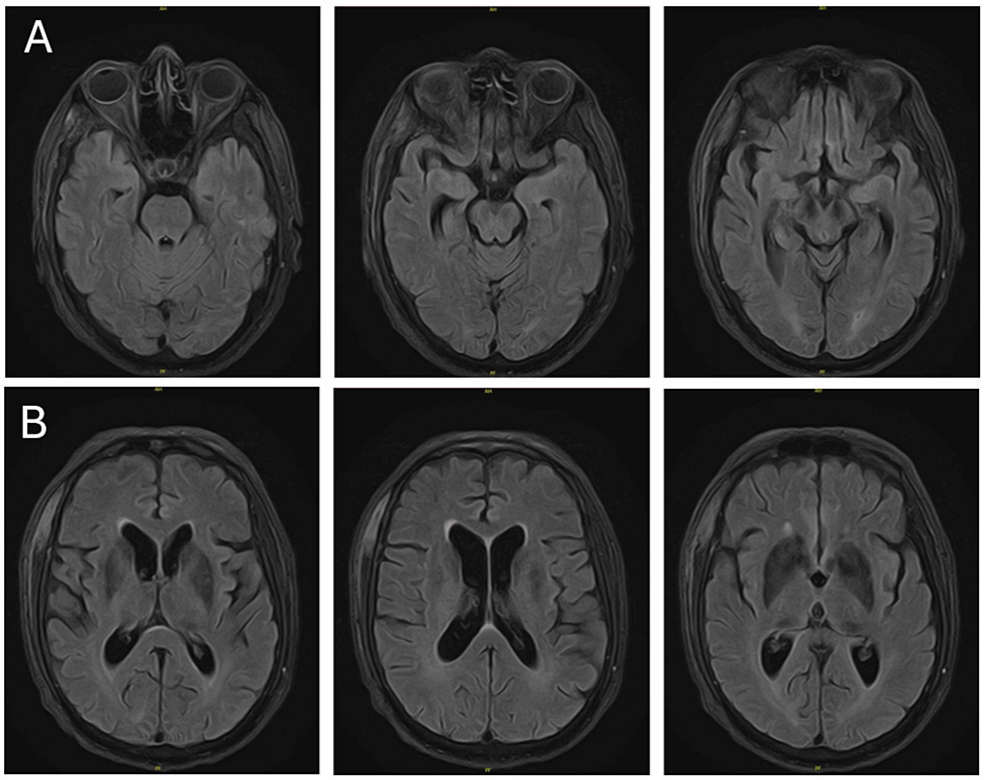
Axial T2-FLAIR view of the brain MRI with non-specific white matter disease but specifically demonstrating absent signal intensity changes in the hippocampus and the basal ganglia A) Serial axial T2-FLAIR images displaying the hippocampus; B) Serial axial T2-FLAIR images displaying the basal ganglia MRI: magnetic resonance imaging; FLAIR: fluid-attenuated inversion recovery

**Figure 2:**
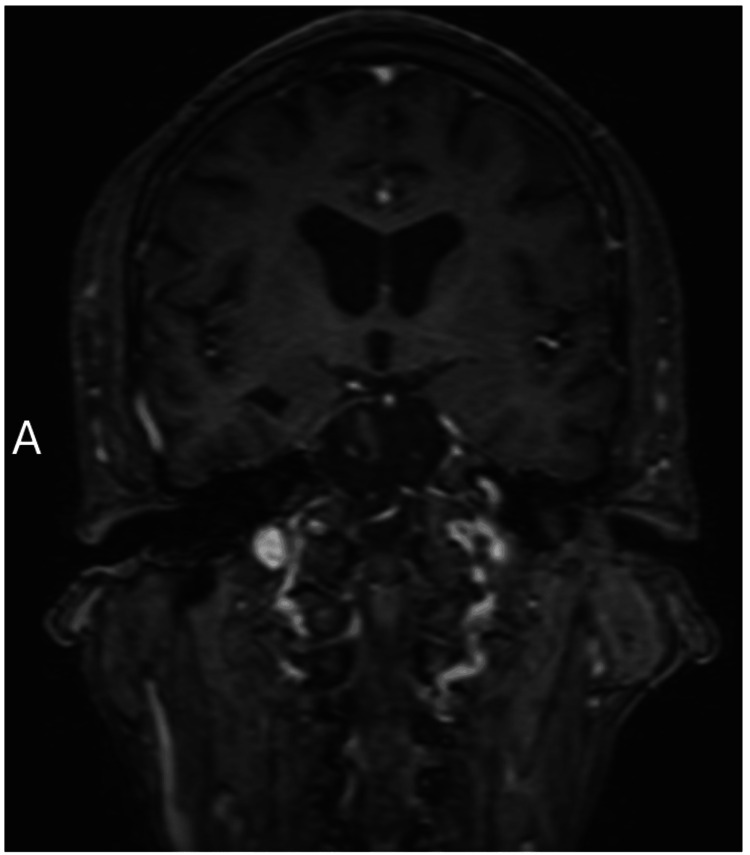
Coronal T2-FLAIR view of the brain MRI demonstrating absent signal intensity changes specifically within the temporal lobe MRI: magnetic resonance imaging; FLAIR: fluid-attenuated inversion recovery

A CT of the head was completed, which showed no acute processes, and a brain MRI with and without contrast was unrevealing with only mild generalized cerebral volume loss and chronic microvascular ischemic changes. Specifically, there were no T2 lesions, contrast-enhancing lesions, or significant hyperintensity in the medial-temporal region or the basal ganglia (Figure 1 and Figure 2). His initial EEG was completed, and it detected signs indicating faciobrachial dystonic seizures and diffuse moderate slowing.

The patient continued to have episodes of both generalized tonic-clonic seizures and faciobrachial myoclonus. Subsequent reads on the continuous EEG further revealed left frontal temporal seizures that persisted despite the patient’s current anti-seizure medications of Keppra and Vimpat. In response, Depakote and phenytoin were added in a stepwise approach.

Though a lumbar puncture was warranted, multiple factors contributed to the delay in the procedure. This included the persistent, recurrent seizures and the poor mental status of the patient among other aspects. Ultimately, the patient was required to be anesthetized for the procedure. A lumbar puncture was finally performed around day 7 of the patient's hospital admission, and then the patient was started on empiric plasma exchange therapy (PLEX). His initial CSF findings showed a protein level of 29, slightly elevated glucose of 100, a total nucleated cell count of less than 5, and negative for any bacterial or viral growth. High-dose steroid therapy was avoided because the patient had a urinary tract infection that was being treated on initial hospital admission.

After five sessions of PLEX, the patient began recovering from his admitted symptoms. His sleep improved, and his family reported a return to his baseline behavior with the resolution of his encephalopathy. With the initiation of PLEX, his FBDS dramatically decreased in frequency by the time of discharge. The autoimmune encephalitis panel returned and revealed positive anti-LGI1 IgG antibodies. He was diagnosed with anti-leucine-rich glioma-inactivated 1 limbic encephalitis and started on rituximab for immunotherapy at discharge. On subsequent follow-up, his seizures were stable on his immunotherapy and anti-seizure therapy that was optimized during the hospital course.

## Discussion

In this case, the patient went for years with progressive neuropsychological symptoms due to his anti-LGI1 limbic encephalitis that was not detected earlier due to fluctuating and nonspecific symptoms and normal MRI imaging. In retrospect, his presenting symptoms should have been key evidence for autoimmune encephalitis, anti-LGI1 LE specifically, and a lumbar puncture should have been initiated earlier to start appropriate management and avoid this patient’s lengthy disease course. This case presents a peculiar and unusual situation because of the benign matter of his MRI findings, despite the profound and progressive symptoms from the patient’s disease over a chronic course. This patient presented with a challenging presentation, as it is expected that with a long history of FBDS and psychiatric symptoms, some lesions should have been seen on imaging.

Recognizing the anti-LGI1 type from other autoimmune encephalitides is complicated in that patients can present with unique and non-specific symptoms. Anti-LGI1 LE is considered to have an acute phase that displays the classic limbic encephalitis findings and a prodromal phase, which consists of FBDS with or without sleep and behavior changes [5]. Anti-LGI1 LE has several symptoms that can distinguish it from other forms of autoimmune encephalitis, including hyponatremia, various seizure semiologies (more specifically the faciobrachial dystonic seizures), and various neurocognitive issues [6]. The seizures experienced by patients are found to vary and may range from subclinical to motor to sensory [7]. A sequela from a sensory seizure or a Todd's paralysis phenomenon could have explained the stroke-like sensory and motor deficits the patient in this case experienced early during his disease course. Faciobrachial dystonic seizures are sudden and brief contractions of the upper limb and, usually, the ipsilateral face [8]. These movements are not restricted to dystonia and can be tonic, clonic, or myoclonic as well. These may have been the earliest signs of the development of FBDS. Due to the myoclonic nature of these movements, the name “faciobrachial dystonic seizures” may be misleading and the more inclusive “faciobrachial motor seizures” may be more fitting [9]. In this case, the patient was noted to have unexplained spasms and jerks of the face and upper limb earlier that were left undiagnosed and untreated. These may have been the earliest signs of the development of FBDS.

It is important to recognize FBDS in limbic encephalitis, as regular anti-seizure medications are less efficacious to control seizures. It has been shown that high-dose steroids, PLEX, or IVIG with long-term DMARDs are the better option in the management of FBDS compared to general anti-seizure medications [10]. Recently cases have been reported with good outcomes utilizing plasma exchange treatment in autoimmune encephalitis [11]. When compared to corticosteroid and IVIG treatment, plasma exchange has been shown to have significantly better outcomes without notable changes in adverse events [12].

Several diagnostic approaches are recommended for anti-LGI1 LE with no consensus on the best approach. Though there are ongoing debates on the sensitivity of diagnostic testing, specifically antibodies from serum vs. cerebral spinal fluid, it has been shown that patients with antibodies positive for LGI1 in the cerebral spinal fluid have poorer outcomes [13,14]. Hyponatremia is another finding that is different than other autoimmune encephalitides. Recent studies have shown that up to 87% of patients with anti-LGI1 LE will develop hyponatremia over the course of the disease. Hyponatremia is thought to be from a resulting SIADH from the inflammatory process of the encephalitis to the hypothalamus [15]. This significant correlation of hyponatremia with anti-LGI1 LE has prompted discussions advocating for the testing of autoimmune encephalitis in cases of patients presenting with seizures and subsequently being found to be hyponatremic [16].

Imaging is also a heavily utilized diagnostic test, as a majority of autoimmune encephalitides follow similar patterns. The mesial temporal lobe and basal ganglia are the two most prominent structures affected by autoimmune encephalitides including anti-LGI 1 LE. The lesions at these structures are detected on MRI as hyperintensities on T2 with edema in the acute phase and can progress to hypointensities with atrophy. The percentage of anti-LGI 1 LE cases with positive MRI findings varies but has been observed as high as 96% in cases with seizure, with the intensity of these lesions being found to correlate to the severity of the disease [5]. In this case, it was unexpected for a patient with his severe presentation and lengthy disease course to have a normal MRI.

The discussion of this case is important, as it relays the importance for providers to not solely rely on imaging. A comprehensive approach is necessary, and if symptoms persist or progress, clinical correlations should warrant an extended workup. Though a lumbar puncture isn’t necessary in every situation, it is an important diagnostic procedure that a neurologist should be quick to proceed with. The correct diagnosis for our presenting patient was late, resulting in a delay in initiating the appropriate management and treatment until the disease had progressed further.

## Conclusions

Anti-leucine-rich glioma-inactivated 1 limbic encephalitis is a young diagnosis and understanding the disease and pathology continues to develop and evolve. Our case underscores the diagnostic challenges associated with anti-LGI1 LE, particularly when a patient presents with vague symptoms and the conventional brain imaging returns unremarkable. When a patient presents with such frank symptoms, the typical MRI findings of lesions in the mesial temporal lobe or basal ganglia are expected, which can make diagnosing anti-LGI1 LE more difficult when imaging is normal. The case also highlights the varied and nonspecific symptoms associated with anti-LGI1 LE, including faciobrachial dystonic seizures, hyponatremia, sleep disturbances, and various cognitive and psychiatric dysfunctions. Recognizing these early signs, along with reassessing the terminology associated with seizures, can help expedite diagnosis and proper intervention of anti-LGI1 LE. This, in turn, can assist in mitigating the prolonged disease course often encountered by patients with anti-LGI1 LE and ineffective treatment regimens given due to these vague and non-specific symptoms.
